# Supplementary material for: Self-reported abortion experiences in Ethiopia and Uganda, new evidence from cross-sectional community-based surveys
Source: PLOS Glob Public Health. 2023 Sep 8;3(9):e0002340. doi: 10.1371/journal.pgph.0002340 (PMC10490852; doi:10.1371/journal.pgph.0002340)
Supplement: S1 Text — Sampling Strategies for Each Survey; Additional information on the Network Scale-Up Method analyses. (DOCX) [file pgph.0002340.s001.docx]

**Supplemental Materials:** **Self-reported abortion experiences in Ethiopia and Uganda, new evidence from cross-sectional community-based surveys**

**Sampling strategies for each survey**

***Female Questionnaire Ethiopia:*** The Ethiopia female questionnaire data was collected in March 2020 as part of a larger panel study investigating the impact of the Global Gag Rule.[1,2] The panel was constructed using baseline data from the 2018 Performance Monitoring for Accountability (PMA) platform.[3] The 2018 PMA survey used a two-stage cluster sample design, with urban-rural statistical regions as the strata, resulting in a nationally representative collection of 221 enumeration areas (EAs). Next, interviewers mapped and listed all households within an EA, and 44 households were randomly selected to participate in the survey. All women aged 15–49 who slept in the household the previous night or were usual members of the household were invited to participate in the survey. For the 2020 survey, respondents were eligible to be reinterviewed if they lived in one of the six most populous regions in Ethiopia (Tigray, Afar, Amhara, Oromiya, SNNPR, and Addis Ababa), resided in the same kebele (or sub-city in Addis Ababa) in 2020 as they did in 2018, and had provided consent in 2018 to be recontacted for future research (n=6306). Approximately 78% (n=4909) of eligible respondents from the 2018 survey were successfully interviewed in 2020. Respondents who were lost to follow-up were more likely to be younger, never married, have completed more schooling, live in a richer household, and reside in an urban area.

***Female Questionnaire Uganda:*** The 2019 PMA survey was designed to be nationally representative. To identify the sample for the female questionnaire, study staff first attempted to re-interview all women who participated in the 2018 PMA survey. Similar to the Ethiopia sampling design, the Uganda 2018 sampling plan relied on a two-stage cluster sampling design, using urban-rural and 10 major administrative regions as the strata, resulting in a nationally representative collection of 110 enumeration areas (EAs) or EA clusters. Interviewers mapped and listed all households within an EA or EA cluster and 44 households were randomly selected. All women aged 15-49 who slept in the household on survey night or were usual members of the household were invited to participate in the female survey. A total of 4,288 women were interviewed in 2018, and approximately 96% (n=4,095) consented to be re-contacted for the 2019 survey.

Women who moved to another household in the same EA between survey rounds were eligible for interview during follow-up. Women who moved outside of the EA were considered lost-to-follow-up. To ensure that the sample was nationally representative in 2019, all households within each EA were mapped and listed, and the 44 households that were randomly sampled in 2018 were re-interviewed. If one of these dwelling of the household was no longer a dwelling or destroyed at the time of 2019 mapping, the household was replaced with another randomly selected household in the EA that was not part of the original 44 households sampled in 2018. This process resulted in a total of 4,586 surveys in 2019 (n=2,755 who were re-located and re-interviewed, n=1,831 women newly sampled and surveyed).

**Additional information on the Network Scale-Up Method analyses**

*Known Population Approach for estimating social network sizes*

The ‘known population’ approach for estimating social network sizes involves asking each respondent to report the number of people she knows who have a certain characteristic for which the true population size of people with that characteristic is “known”.[4,5] In this study, known population sizes were determined using data from the most recent Demographic and Health Survey (DHS) in each country in combination with World Population Prospects (WPP) data on the population of women aged 15-49 in 2020 in Ethiopia and 2019 in Uganda. For example, using data from the 2016 Ethiopian DHS and 2020 WPP estimates, we estimate that approximately 539,000 women aged 15-49 live in a household that owns a camel in Ethiopia. In our survey, we ask respondents to report the total number of women they know who live in a household that owns a camel. If a respondent says she knows 2 women who fit this characteristic, we estimate that the respondent knows 2 out of 539,000 women of reproductive age in Ethiopia. We then multiply 2/539,000 by 26,725,476 (the total number 15–49-year-old living in Ethiopia in 2020) to get an estimate of her social network size. In practice, social network sizes are not estimated with only one known population. Instead, we estimate network sizes with data from multiple known population questions using the following formula:

$$\hat{c_{i}}=\frac{\sum_{j} m_{ij}}{\sum_{j} e_{j}}*t$$

where $\hat{c_{i}}$ is the estimated social network size of respondent *i*, $m_{ij}$ is the number of people in known population *j* that respondent *i* knows, $e_{j}$ is the size of known population *j*,and *t* is the size of the general population.[5,6]

To increase respondents’ ability to accurately report the number of women they know with each characteristic, we selected populations based on characteristics that are visible and likely known by respondents (i.e. women who gave birth in the past year). In addition, the characteristic should be rare enough for respondents to accurately count all members of her social network with that characteristic. (Ideally, known populations should represent less than 5% of all women aged 15-49.) To prevent outlier responses from unduly biasing social network size estimates, we top-coded all responses to the known population questions at 30. This technique has been used in a number of previous NSUM studies.[7–11]

We included 13 known population questions in the Ethiopian female questionnaire and 12 in Uganda. (See Table A.) While we attempted to limit population selections to those that met the two criteria listed above, limited data availability meant that some of our known population represented more than 5% of all women aged 15-49. In order to further ensure the accuracy of reported known population sizes, we excluded known populations in the analysis phase if more than 2% of the respondents reported knowing 30 or more people in that population. This is resulted in the exclusion of 2 known populations in Ethiopia and 1 in Uganda.

*Internal Validity Checks*

We test how the NSUM performed in estimating social network sizes using an internal validity check; we remove one known population at a time, treated that known population as if we do not know its size, and use the same NSUM procedures described above to estimate the size of the removed population [12]. The closer this newly estimated population size is to the actual known size of the population, the more likely it is that the NSUM is accurately estimating the size of respondents’ social networks. As such, back-estimates that are 100% of the known population size are an indicator of perfect performance, and previous studies have used indicated that back-estimates between 50-200% of the known population size are considered to be performing suitably well [13–15].

*Calculating the one-year induced abortion incidence estimate*

Respondents are asked to report the number of women in their social networks who had an induced abortion within the past year. This number is used in combination with the personal network size estimates to estimate the total number of women who have had an abortion in each country using the following formula:

$$\hat{e}=\frac{\sum_{i} (m_{ij}*\pi_{i})}{\sum_{i} (\hat{c}_{i}*\pi_{i})}*t$$

where $\hat{e}$is the estimated number of women who had an induced abortion, $m_{ij}$ is the number of women that respondent *i* knows with characteristic *j* (induced abortion),$\pi_{i}$ is the inverse probability of selection for respondent *i*, $\hat{c_{i}}$ is the estimated social network size of each respondent *i*, and *t* is the size of the population of women aged 15-49 in each country.[5,6]

To produce a certainty interval for the NSUM estimates of induced abortion incidence, we use a rescaled bootstrap variance estimation procedure.[16] Ninety-five percent certainty intervals are estimated from a set of 5,000 replicate samples generated through the rescaled bootstrapping technique.

*Results*

Average network sizes were 29.4 (range: 0-318.5) in Ethiopia and 17.8 (range: 0-195.7) in Uganda (Figure 1). Our validity checks show that the DHS population sizes and NSUM estimates are relatively close (Table 1). The NSUM performed slightly worse in Ethiopia compared to Uganda, where two known populations were substantially overestimated (women who live alone, 595% of DHS estimate; women who live in a household that owns a motorcycle, 446% of DHS estimate.) However, the remaining 9 known population estimates were within acceptable bounds. In Uganda, there was only one known population whose back-estimation fell outside of the 50%-200% bound (women with a husband/partner who does not work, 279.8% of DHS estimate), and we did not see evidence of consistent over- or under-estimation.

Table 2 displays the unadjusted NSUM abortion incidence estimates, which are 4.7 per 1000 women aged 15-49 in Ethiopia (95% CI 3.9-5.6) and 19.4 per 1000 (95% C 16.2-22.8) in Uganda. These estimates do not account for transmission bias, which is the phenomenon that respondents will not be aware of all abortions that occur within their social networks.[17,18] As such, these estimates should be considered as minimum values; the true abortion incidence rate in each country is higher. The magnitude of this delta between the estimate minimum incidence rate and the true rate is dependent on how visible abortions are within broad social networks.[19]

***
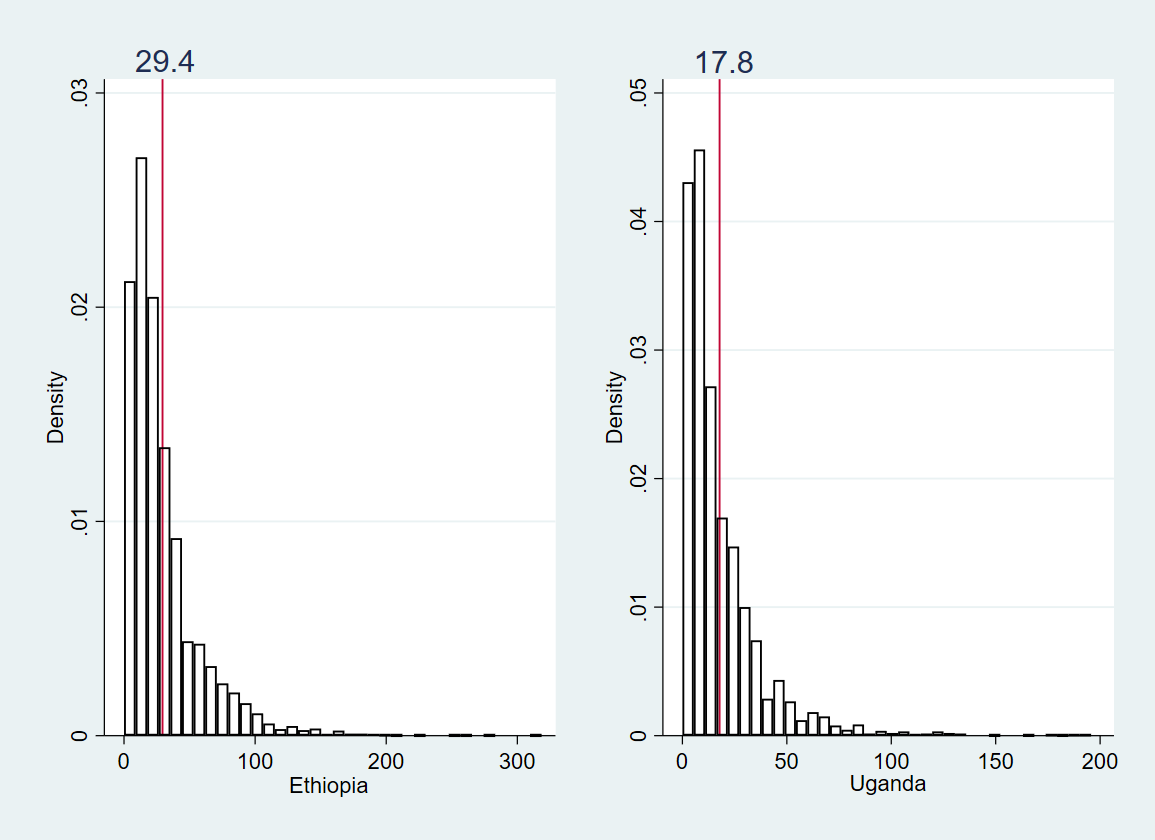
***

**Fig A. Degree distribution from NSUM in Ethiopia and Uganda**

**Table A. Internal validity check results comparing NSUM population back-estimates to DHS population sizes, Ethiopia and Uganda**

| **Category of population** | **DHS Size** | **Mean number of connections** | **Mean number of connections,**  **top-coding at 30** | **Initial NSUM estimate (as % of DHS estimate)** |
| --- | --- | --- | --- | --- |
| **Known populations used in Ethiopia, women ages 15-49*** | | | |  |
| Gave birth in last 12 months | 4,335,398 | 4.7 | 4.5 | 104.8% |
| Most recent birth was a multiple birth | 274,902 | 0.3 | 0.3 | 110.4% |
| Works as a cleaner | 806,794 | 2.0 | 1.9 | 149.3% |
| Lives alone | 450,005 | 3.0 | 2.8 | 592.5% |
| Smokes a pipe or cigarettes | 165,682 | 0.3 | 0.3 | 116.5% |
| Has at least one co-wife | 2,689,014 | 1.3 | 1.2 | 50.2% |
| Lives in a household: |  |  |  |  |
| ...that owns a beehive | 3,363,962 | 1.3 | 1.3 | 49.1% |
| ...that owns a computer | 901,877 | 1.3 | 1.1 | 62.4% |
| ...that owns a scooter or motorcycle | 341,059 | 1.5 | 1.5 | 446.2% |
| ...that owns a bicycle | 732,308 | 0.6 | 0.6 | 59.6% |
| ...that owns at least one camel | 538,923 | 0.5 | 0.4 | 64.9% |
| **Known populations used in Uganda, women ages 15-49**** | | | |  |
| Gave birth in last 12 months | 1,866,029 | 3.6 | 3.4 | 114.2% |
| Husband/partner does not work | 211,554 | 1.3 | 1.1 | 279.8% |
| Has at least one co-wife | 1,542,103 | 2.8 | 2.7 | 92.3% |
| Attended any education past senior six | 793,764 | 1.8 | 1.5 | 108.9% |
| Lives in a household: |  |  |  |  |
| …that owns a motorcycle or scooter | 1,203,259 | 1.8 | 1.8 | 94.8% |
| ...that owns a car or truck | 587,313 | 1.2 | 1.1 | 105.0% |
| ...that has a refrigerator | 777,497 | 1.2 | 1.1 | 77.1% |
| ...that owns an exotic cow | 410,925 | 0.7 | 0.7 | 97.8% |
| ...that owns at least one sheep | 806,281 | 1.2 | 1.2 | 74.9% |
| ...that has a landline | 134,188 | 0.1 | 0.1 | 65.5% |
| Sources:  *2016 Ethiopia DHS, female (FQ) and household (HHQ) questionnaires  **2016 Uganda DHS, female (FQ) and household (HHQ) questionnaires | | | |  |

**Table B. NSUM minimal annual abortion incidence estimates for Ethiopia and Uganda**

|  | Rate per 1000 | 95% CI | |
| --- | --- | --- | --- |
| Ethiopia annual abortion incidence rate | 4.7 | 3.9 | 5.6 |
| Uganda annual abortion incidence rate | 19.4 | 16.2 | 22.8 |

**References**

1. Sully E, Shirefaw S, Seme A, Bell S, Giorgio M. Impact of the Trump Administration’s expanded Global Gag Rule policy on family planning service provision in Ethiopia. Studies in Family Planning. 2022; Forthcoming.

2. Sully E, Shiferaw S, Seme A, Bell SO, Chiu DW, Giorgio M. The Impact of the Global Gag Rule on Women’s Contraceptive Use and Reproductive Health Outcomes in Ethiopia. Under Review. 2022.

3. PMA. Summary of the sample design for PMA2016/Ethiopia-R6. In: PMA2018/Ethiopia Round 6 Indicators [Internet]. 2018 [cited 25 Sep 2020]. Available: https://www.pmadata.org/countries/ethiopia/ethiopia-indicators/pma2018ethiopia-round-6-indicators

4. Killworth PD, McCarty C, Bernard HR, Shelley GA, Johnsen EC. Estimation of Seroprevalence, Rape, and Homelessness in the United States Using a Social Network Approach. Eval Rev. 1998;22: 289–308. doi:10.1177/0193841X9802200205

5. McCarty C, Killworth PD, Bernard HR, Johnsen EC, Shelley GA. Comparing Two Methods for Estimating Network Size. Human Organization. 2001;60: 28–39. doi:10.17730/humo.60.1.efx5t9gjtgmga73y

6. Bernard H, McCarty C. The Network Scale-Up Method: Background and Theory. 2009. Available: http://nersp.osg.ufl.edu/~ufruss/scale-up/scale-up%20method%20theory%20and%20history%20with%20notes.pdf

7. Sully E, Giorgio M, Anjur-Dietrich S. Estimating Abortion Incidence Using the Network Scale-up Method. Demographic Research. 2020;43: 1651–1684.

8. McCormick T, Salganik M, Zheng T. How many people do you know?: Efficiently estimating personal network size. J Am Stat Assoc. 2010;105: 59–70. doi:10.1198/jasa.2009.ap08518

9. RBC/IHDPC. Estimating the Size of Populations through a Household Survey. Calverton, Maryland, USA: Rwanda Biomedical Center/Institute of HIV/AIDS, Disease Prevention and Control Department (RBC/IHDPC), School of Public Health (SPH), UNAIDS, and ICF International; 2012. Available: https://dhsprogram.com/pubs/pdf/FR261/FR261.pdf

10. Salganik M, Fazito D, Bertoni N, Abdo A, Mello M, Bastos F. Assessing Network Scale-up Estimates for Groups Most at Risk of HIV/AIDS: Evidence From a Multiple-Method Study of Heavy Drug Users in Curitiba, Brazil. American Journal of Epidemiology. 2011;174: 1190–1196. doi:10.1093/aje/kwr246

11. Zheng T, Salganik M, Gelman A. How Many People Do You Know in Prison?: Using Overdispersion in Count Data to Estimate Social Structure in Networks. Journal of the American Statistical Association. 2006;101: 409–423. doi:10.1198/016214505000001168

12. Feehan D, Umubyeyi A, Mahy M, Hladik W, Salganik M. Quantity Versus Quality: A Survey Experiment to Improve the Network Scale-up Method. Am J Epidemiol. 2016;183: 747–757. doi:10.1093/aje/kwv287

13. Guo W, Bao S, Lin W, Wu G, Zhang W, Hladik W, et al. Estimating the Size of HIV Key Affected Populations in Chongqing, China, Using the Network Scale-Up Method. PLOS ONE. 2013;8: e71796. doi:10.1371/journal.pone.0071796

14. Habecker P, Dombrowski K, Khan B. Improving the Network Scale-Up Estimator: Incorporating Means of Sums, Recursive Back Estimation, and Sampling Weights. PLOS ONE. 2015;10: e0143406. doi:10.1371/journal.pone.0143406

15. Kadushin C, Killworth P, Bernard H, Beveridge A. Scale-Up Methods as Applied to Estimates of Heroin use. Journal of Drug Issues. 2006;36: 417–440. doi:10.1177/002204260603600209

16. Feehan DM. surveybootstrap: Tools for the Bootstrap with Survey Data. R package version 0.0.1. 2016. Available: https://CRAN.R-project.org/package=surveybootstrap

17. Bernard HR, Hallett T, Iovita A, Johnsen EC, Lyerla R, McCarty C, et al. Counting hard-to-count populations: the network scale-up method for public health. Sexually transmitted infections. 2010;86: ii11–ii15.

18. Killworth PD, McCarty C, Johnsen EC, Bernard HR, Shelley GA. Investigating the Variation of Personal Network Size Under Unknown Error Conditions. Sociological Methods & Research. 2006;35: 84–112. doi:10.1177/0049124106289160

19. Salganik M, Mello M, Abdo A, Bertoni N, Fazito D, Bastos F. The Game of Contacts: Estimating the Social Visibility of Groups. Soc Networks. 2011;33: 70–78. doi:10.1016/j.socnet.2010.10.006
