# Supplementary material for: Self-reported abortion experiences in Ethiopia and Uganda, new evidence from cross-sectional community-based surveys
Source: PLOS Glob Public Health. 2023 Sep 8;3(9):e0002340. doi: 10.1371/journal.pgph.0002340 (PMC10490852; doi:10.1371/journal.pgph.0002340)
Supplement: Attachment — Submitted filename: Self-reported abortion_response to reviewers.docx [file pgph.0002340.s003.docx]

**Self-reported abortion experiences in Ethiopia and Uganda, new evidence from cross-sectional community-based surveys**

**Response to Reviewers**

| **Editor Comments** |  |
| --- | --- |
| Please include a complete copy of PLOS’ questionnaire on inclusivity in global research in your revised manuscript. Our policy for research in this area aims to improve transparency in the reporting of research performed outside of researchers’ own country or community. The policy applies to researchers who have travelled to a different country to conduct research, research with Indigenous populations or their lands, and research on cultural artefacts. The questionnaire can also be requested at the journal’s discretion for any other submissions, even if these conditions are not met.  Please find more information on the policy and a link to download a blank copy of the questionnaire here: <https://journals.plos.org/plosone/s/best-practices-in-research-reporting>. Please upload a completed version of your questionnaire as Supporting Information when you resubmit your manuscript. | We have completed this questionnaire and included it in the resubmission. |
| Please provide separate figure files in .tif or .eps format. | We have uploaded the figures separately and in the proper format. |
| Please review your reference list to ensure that it is complete and correct. If you have cited papers that have been retracted, please include the rationale for doing so in the manuscript text, or remove these references and replace them with relevant current references. Any changes to the reference list should be mentioned in the rebuttal letter that accompanies your revised manuscript. If you need to cite a retracted article, indicate the article’s retracted status in the References list and also include a citation and full reference for the retraction notice. | We have reviewed the reference list, and no changes have been made due to retractions or updated sources. However, we have added a few additional references based on the reviewer comments. |
| **Reviewer 1 Comments** |  |
| The manuscript is written in a clear and understandable language, however there are still some mistakes that persist in the text, as well as abbreviations that are cited without having been spelled beforehand. The correction of all these typos would improve the quality of the final manuscript. | We thank the reviewer for this comment. We have carefully reviewed the manuscripts to fix mistakes and appropriately spell out abbreviations. |
| 231-233:: ‘Next, we use chi-squared tests and t-tests to investigate bivariate differences in these characteristics based on whether women self-reported ever having an abortion.’ The authors should specify in which case they used one or the other test, and the conditions of applicability of the statistical model (p-value, confidence interval). This will help to better understand the rigor of their analysis and the accuracy of their interpretation of the results. | We thank the reviewer for this comment. We modified the text to provide more clarity on our statistical testing: “Next, we used chi-squared tests and t-tests to investigate bivariate differences in women’s self-report of ever having an abortion for categorical and continuous characteristics, respectively, using a p-value cutoff of 0.05 to determine statistical significance.” We have also added notes in Table 1 indicating which tests were used to determine statistical significance.  We also provide 95% confidence intervals around the abortion incidence estimates displayed in Figures 1 and 2, to illustrate the precision of our estimates. |
| 286-288: ‘In both countries, there were statistically significant differences in all socio-demographic characteristics between women who self-reported an induced abortion and those who did not’ As noted above, the lack of precision on the significance level does not allow us to verify the authors' interpretation of the results. Indeed, in Table 1, the p-values for the variables 'Education' and 'Residence' for Uganda leave us in doubt. | We thank the reviewer for catching this issue and apologize for the mistake. The differences are statistically significant in the unweighted data. However, we decided to display the weighted data in this table and did not update the text accordingly. We have revised the text to fix this error. In relation to the urban/rural difference in Uganda, given that the pattern across the two countries remains consistent and that the p-value is approaching significance (p=0.055), we note in the paper that the difference by urban/rural in Uganda is marginally statistically significant. |
| 290-294: ‘In addition, larger proportions of women who self-reported abortion had a secondary education or greater (Ethiopia: 36.0% vs. 24.2%; Uganda: 43.8% vs. 34.9%), lived in urban areas (Ethiopia: 62.4% vs. 22.7%; Uganda: 32.9% vs. 22.8%), were formerly married (Ethiopia: 22.6% vs. 10.5%; Uganda: 29.2% vs. 13.2%), or had any children (Ethiopia: 88.0% vs. 71.9%; Uganda:88.5% vs. 74.9%).’ The authors should interpret the results as presented in Table 1. Indeed, the variable "education" is categorized into 4 modalities and not 2, which confuses the interpretation of the authors for the reader. | We appreciate the reviewer’s concern and agree that the original text was somewhat confusing. In the interest of brevity, we collapsed the highest two education categories in the text, which was confusing for the reader. Since a small proportion of respondents are in the highest education category in both countries, we have revised Table 1 and the associated text so that the top 2 education categories are collapsed, consistent with the text in the results section. |
| 326-329: ‘Of the remaining abortions, 14.4% were less safe and 23.2% were least safe. Abortions that resulted in a treated complication were more commonly reported among least and less safe abortions compared to safe abortions (21.4% and 23.1% vs. 12.4%, respectively; Figure 1).’ The authors should discuss these results a little more, as they highlight the quality of care received in the health facilities, in a context where the practice is legal. In addition to strengthening the structural capacity of health facilities, the availability of management protocols, equipment and inputs, and qualified and motivated staff should be ensured. | We appreciate this comment from the reviewer. However, we are concerned about drawing conclusions about quality of care from health facilities for several reasons. First, this study was not designed to measure quality of care, and standard indicators that would better assess quality are not available. In addition, self-reported treated complications are not a great proxy for the severity of these complications. In many cases, women may be seeking care after they experience bleeding that is expected after taking medication abortion, but they were not actually experiencing a complication. Finally, we do not feel confident drawing conclusions about quality of care based on the limitations of our safety indicator. As we outline in the discussion, we do not have detailed information on the exact procedures and abortion methods, which may have been inaccurately reported. Further, our assumption that all facility-based abortions were performed by trained providers may be incorrect. Misclassification for either of these indicators could bias our safety estimates. As such, its possible that the 12.4% of “safe” abortions that resulted in a complication that required treatment may be a reflection of the imprecision of our safety variable, not concerns around quality of care. |
| 352-354 ‘We found that women who self-reported an induced abortion differed systematically from women who did not, with women who self-reported abortions in both countries being older, more educated, with more children, as well as less likely to be married or live in a rural area.’ As noted above, the limited information provided by the authors makes it impossible to agree with them on these conclusions, and it would be risky to make recommendations based on such an interpretation. | We have revised the text that the reviewer highlighted in this comment to reflect the fact that differences by education level were not statistically significant in Uganda. |
| 408-412 ‘In the context of Ethiopia, where abortion is already legally accessible, it may be important for donors and service providers to consider investments in safe abortion care services in the private sector as a complement to the government’s continued focus on strengthening the infrastructure of public sector abortion services. (6,9,38) This will help ensure greater access to quality abortion care services that meets women’s preferences and needs.’ We are concerned that donors’ emphasis on private health facilities will contribute to inequities and inequalities in access to safe abortion services. It would be interesting to investigate why, despite the broad legal context, women prefer to use private services and try to solve these problems for a wider and more equitable access | We appreciate this comment from the reviewer, and we absolutely do not want to contribute to inequities in access to safe abortion care. That said, it is also important to address the needs and preferences of women in the short-term. Further we have also learned that, for a wide range a health services, we need a diverse range of sources of care across both the public and private sector in order to meet the patient needs.  In response to this comment, we have revised the corresponding text in the discussion to incorporate the reviewer’s suggestion to conduct research on why women may prefer to use private providers, the results of which can be used to help improve care in the public sector. After this addition, we still include our comment suggesting investments in the private sector as a complement to the suggestion above. However, we removed the language targeting donors specifically. |
| **Reviewer 2 Comments** |  |
| - Self-reported data have inherent limitations and potential sources for bias. For this, the authors are not to be blamed. In fact they should be congratulated for recognizing, acknowledging and discussing them. | We thank the reviewer for this comment. We wanted to be clear about the biases inherent in this data. However, we still believe that it can provide some helpful information about induced abortion in each context. |
| - The study provides a wealth of information. It may be important to highlight findings that are most relevant to health policymakers, and let them stand out clearly. Two findings deserve particular emphasis and are relevant when two countries with different abortion legal contexts are compared and it would be good to have then highlighted in the abstract, discussion and conclusions sections: -Self-reported treated post-abortion complications were more than twice as high in Uganda where abortion is restricted (37.2% ) compared to 16.0% in Ethiopia. -Estimated abortion incidence was much higher in Uganda where it is legally restricted. The minimum one year abortion incidence rate was estimated as 4.7 per 1000 in Ethiopia and 19.4 in Uganda | We appreciate this comment from the reviewer. To the first point, we discuss this finding in detail in the discussion (paragraph that begins on line 444), noting that this difference may be explained by the underlying legal differences in abortion access between the two countries.  Our conclusion also focuses on the key policy take-aways from our paper, including the high rate of contraceptive non-use prior to the pregnancy for which they had an abortion, and the higher rates of complications and unsafe abortions in Uganda, the more legally restricted context.  Focusing on this second point, we want to be careful about how we interpret the incidence findings. As the reviewer notes, these are minimum estimates. While it is interesting that the minimum estimate in Uganda is much higher than in Ethiopia, this could reflect reporting patterns instead of true underlying differences in the incidence rates in the two countries. Given the limitations of the study data and the limited information of the size of transmission bias in each country, we purposely do not try to compare the two estimates to one another. This is why we instead emphasize the utility of minimum estimate for global modeling efforts. |
| - Procedure safety (as defined for public health purposes) is no more important than actual patient safety as reported complications. Patient safety data deserve more emphasis and detail. | We thank the reviewer for this comment, and we absolutely agree. We have revised the discussion section to highlight this limitation of the Ganatra safety definition. Unfortunately, our only data on abortion outcomes comes from whether a woman self-reported that her abortion resulted in a complication that required treatment in a health facility. Because we do not have details about the exact nature of the complications or clinical outcomes, it is difficult to interpret these self-reports as exact proxies for unsafe abortions. In some cases, women may experience normal bleeding after a medication abortion but still decide to seek PAC, either because they were not given adequate information on what to expect or because they prefer to complete their abortion within a formal health facility. We also note this limitation in the discussion section. |
| - If data were available in Ethiopia before and after the abortion law was expanded, it would be good to mention in the discussion. | We agree this would be helpful, but unfortunately, we do not have data on safety that is easily comparable from before and after the expansion of the abortion law in Ethiopia. |
| - Reference 4 is not complete. It is one of the good Guttmacher reports. | We apologize for this oversight, and we have updated the reference in the reference list. |
